# Comparing how patients value and respond to information on risk given in three different forms during dental check-ups: the PREFER randomised controlled trial

**DOI:** 10.1186/s13063-019-3824-3

**Published:** 2020-01-06

**Authors:** R. Harris, V. Lowers, L. Laverty, C. Vernazza, G. Burnside, S. Brown, L. Ternent

**Affiliations:** 1 Department of Health Services Research, Institute of Population Health Sciences, University of Liverpool, Room 124, 1st Floor Block B, Waterhouse Building, 1–5 Brownlow Hill, Liverpool, L69 3GL UK; 2 School of Dental Sciences, Newcastle University, Newcastle-upon-Tyne, Tyne and Wear UK; 3 Department of Biostatistics, Institute of Translational Medicine, University of Liverpool, Liverpool, UK; 4 Institute of Health and Society, Newcastle University, Newcastle-upon-Tyne, Tyne and Wear UK

**Keywords:** Risk, Communication, Oral health, Health education, Behaviour change, Dental practice

## Abstract

**Background:**

This study aims to compare patient preference for, and subsequent change in, oral health behaviour for three forms of risk information given at dental check-ups (verbal advice compared to verbal advice accompanied by a traffic light (TL) risk card; or compared to verbal advice with a quantitative light fluorescence (QLF) photograph of the patient’s mouth).

**Methods:**

A multi-centre, parallel-group, patient-randomised clinical trial was undertaken between August 2015 and September 2016. Computer-generated random numbers using block stratification allocated patients to three arms. The setting was four English NHS dental practices. Participants were 412 dentate adults at medium/high risk of poor oral health. Patients rated preference and willingness to pay (WTP) for the three types of information. The primary outcome was WTP. After receiving their check-up, patients received the type of information according to their group allocation. Follow-up was by telephone/e-mail at 6 and 12 months. Mean and median WTP for the three arms were compared using Wilcoxon signed-rank tests. Tobit regression models were used to investigate factors affecting WTP and preference for information type. Secondary outcomes included self-rated oral health and change in oral health behaviours (tooth-brushing, sugar consumption and smoking) and were investigated using multivariate generalised linear mixed models.

**Results:**

A total of 412 patients were randomised (138 to verbal, 134 to TL and 140 to QLF); 391 revisited their WTP scores after the check-up (23 withdrew). Follow-up data were obtained for 185 (46%) participants at 6 months and 153 (38%) participants at 12 months. Verbal advice was the first preference for 51% (209 participants), QLF for 35% (145 participants) and TL for 14% (58 participants). TL information was valued lower than either verbal or QLF information (*p* < 0.0001). Practice attended was predictive of verbal as first preference, and being older. Practice attended, preferring TL the most and having fewer than 20 teeth were associated with increased WTP; and living in a relatively deprived area or having low literacy decreased WTP. There were no significant differences in behaviour change on follow-up.

**Conclusions:**

Although a new NHS dental contract based on TL risk stratification is being tested, patients prefer the usual verbal advice. There was also a practice effect which will needs to be considered for successful implementation of this government policy.

**Trial registration:**

ISRCTN, ISRCTN71242343. Retrospectively registered on 27 March 2018.

## Contributions to the literature

- Although risk stratification strategies are increasingly being used to target anticipatory care efforts, little is known about patients’ perspectives.
- We found that patients preferred risk information given in the form of usual verbal advice, rather than that presented using a traffic light (TL) algorithm. There were also some differences according to the practice attended. This will inform plans to implement a new NHS dental practice contract based on TL risk stratification of patients at initial visits.
- These findings show a gap between a risk stratification policy aimed at promoting preventive care in primary care settings and its value to patients.

## Background

Risk stratification is increasingly used in healthcare as a way to help focus resources and anticipatory care efforts on the people most in need. Although this has been most widely applied in America and in the private sector, its use is increasing in Europe [1]. Accordingly, risk assessments for poor oral health are now incorporated into clinical guidelines for preventive dentistry as well as into a new model for public-sector dental practitioner remuneration in England [2]. This model involves categorising patients into traffic light risk groups at the patients’ dental check-up: Red (high), Amber (medium) and Green (low), which then informs the level of treatment as well as the extent of preventive care the patient receives [3]. The ‘traffic light’ (TL) system can limit patients’ access to advanced restorations such as crowns if their oral health is deemed too poor, so it is also important for patients—although the policy is mainly intended to help standardise preventive care given by practitioners and as a mechanism to transfer at least some responsibility for preventing poor oral health to the patients’ themselves through improving tooth-brushing and dietary habits [3].

A previous review has identified a gap between knowledge and clinical practice in the implementation of risk stratification strategies [1]. This has been previously attributed in part to clinician engagement, for ‘clinicians have to see the point of risk stratification, otherwise it will be really difficult to implement’ [1]. The patients’ view, however, has yet to be explored, although it is possible that if the system is not appreciated by patients, it alters how clinicians incorporate this into their routine practice.

In the process of evaluating the new model contract for dental practitioners in England, eight focus groups of NHS patients were held in four areas of the country, and a questionnaire sent to 200 patients from 70 dental practices piloting the new system across the country, in parallel with a questionnaire to the staff involved [4]. Findings were promising, although there were some differences between patients and staff attitudes. While 41% of patients said their TL rating made no difference to how they looked after their mouths, this compared to 19% as judged by staff; and while over 80% of staff thought it was helpful to be able to show patients their TL status on paper or computer before they left the surgery, only 41% of patients recalled being given the information, with patients from practices at the lower end of the socio-economic spectrum being the least likely to recall being offered/given associated health behaviour advice—even though these patients were the most likely to benefit from improved self-maintenance.

It is therefore important to more fully understand how risk stratification information is received by patients and whether this leads to improvements in health behaviour—and especially whether there are differences between patients according to socio-demographic characteristics. While early pilots of the new NHS dental practice established a system using computer print-outs given to patients, interview data reported this was of limited value to patients: ‘We started to find loads of computer plans in the bin’ [4]. However, the information given in other ways may have more traction, as suggested by studies outside clinical dentistry, where people’s attention and understanding of health education material is found to be enhanced when the information is presented in a vivid way, such as with pictures [5]. Pictures are found to help with both recall and persuasion, with the impact found to be greatest in patients with low literacy [6].

One way of presenting information on the risk of poor oral health is by using photographs of the patients’ mouth. While disclosing dye, which stains where plaque deposits have accumulated around teeth because of inadequate toothbrushing, has been used for many years as a health education tool [7], tele-dentistry is a growing field, with intra-oral cameras now allowing patients to see detailed pictures of their teeth and gums which can be used to reinforce health education advice [8]. A camera system which uses the intrinsic fluorescence of teeth (quantitative light fluorescence (QLF)) is one such technology, which produces a visualisation of early tooth decay as dark areas where the intrinsic fluorescence of teeth is reduced, even before it can be seen by the naked eye [9]. QLF can also highlight plaque which has been present in the mouth for more than 48 h as bright red/orange areas [10]. This presents a modern and vivid way to present information on oral health risk to patients, although previous studies evaluating how patients receive and act on this information have been mainly limited to selected groups of motivated patients such as those receiving orthodontic treatment, involving small samples [11]. Its application as a risk communication tool supporting increased emphasis on preventive care advice in dental practice is as yet untested. A pragmatic trial evaluating how patients’ value and respond to information on their risk of poor oral health given in the context of general dental practice is therefore needed and will significantly contribute to NHS policy involving new dental contract models being piloted, as well as wider developments concerning implementation of risk stratification in other settings.

## Methods

Ethical approval was obtained (number 14/NW/1016). A full description of trial processes has been published in a protocol [12]. The trial aim was to compare how dental patients’ value and respond to information on risk in three ways: usual verbal advice (V); V supported by information on their TL rating; and V supported by a QLF photograph.

This was a multi-centre, parallel-group, patient-randomised RCT undertaken in four multi-surgery dental practices situated in northern England urban areas. In these practices, between 70 and 95% of patients were treated under NHS reimbursement arrangements. Practice 1 was situated in a relatively affluent area where the Index of Multiple Deprivation (IMD) decile was 9 (20% least deprived), whereas Practices 2 and 3 were in very deprived areas (IMD decile 1 = 10% most deprived in the country). Practice 4 was located in IMD decile 2.

### Trial processes and site training

The whole dental team (including receptionists) in the four practices received training related to trial processes (e.g. patient consent, randomisation) as well as study-specific training (giving information in the three forms). Dental nurses were trained to use the QLF camera, and dentists were given information and a crib sheet to help interpret QLF photographs and guide the giving of relevant information to the patient. For example, the crib sheet included a standard message: ‘This red patch on the QLF photograph shows bacteria which have not been cleaned by you for 2–3 days. If you do not improve tooth-brushing here you are highly likely to develop problems.’ Dentists were also trained in the use of the TL oral health risk algorithm, and this was reinforced by providing written guidance (laminated copies) on the risk categorisation system for the whole dental team. All practices received considerable support and training from the research team: for example, one practice received five separate training sessions because of staff turnover.

### Participants

Inclusion criteria were: adults (aged 18+ years) with at least some teeth, identified as Red or Amber risk for poor oral health using the TL algorithm being piloted elsewhere in NHS practices [2, 12]. Exclusion criteria were: patients identified as ‘Green’ (low risk); patients attending for an emergency appointment (because a full check-up and risk assessment is not usual care); and patients with low English language ability who required an interpreter for appointments [12]. Patients were approached to take part by trained dental staff when making an appointment for an NHS dental check-up.

### Randomisation

After enrolment, reception staff randomised patients to one of the three groups by taking sequentially numbered envelopes. Allocation was revealed when patients gave the envelope to the dental team in the surgery to be opened after the check-up had been completed and before oral health advice was given. The allocation sequence was generated by the trial statistician using computer-generated random numbers with a random permuted block size, stratified by practice. A researcher collecting 6-month and 12-month follow-up data on behaviour change by telephone was blind to allocation.

### Intervention

**Fig. 1:**
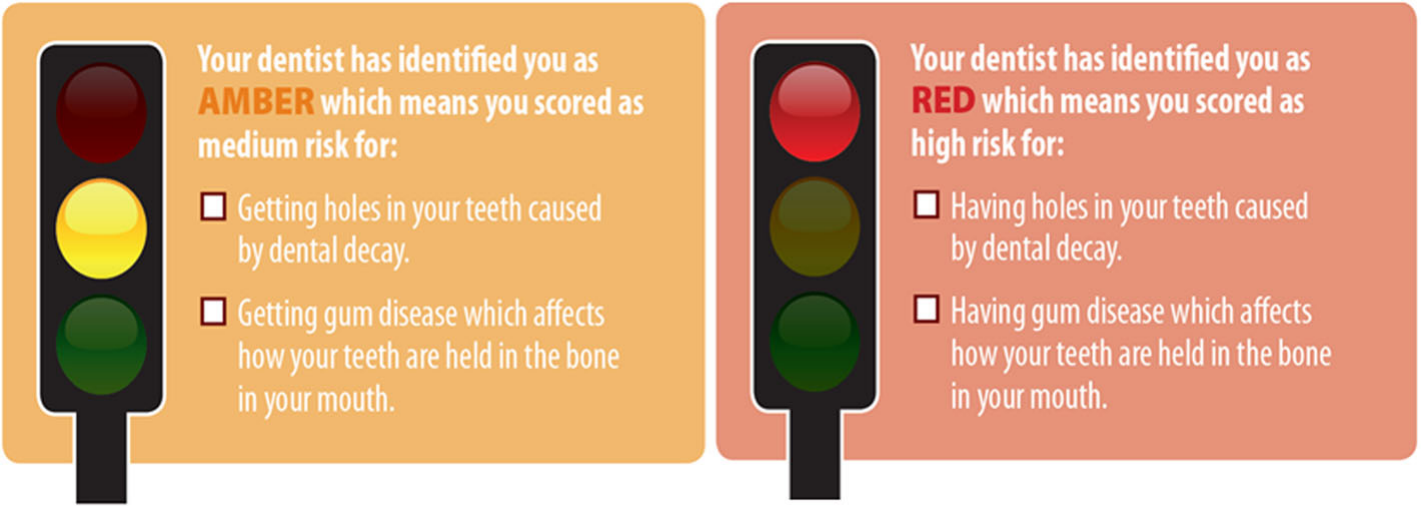
Traffic light intervention cards showing risk information

**Fig. 2:**
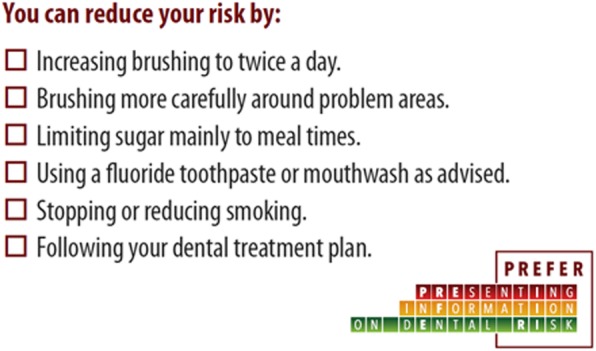
Information on how to reduce risk given to all three groups, including the control (verbal only) group

In the TL arm, dentists gave patients either small Red or Amber risk cards (Fig. 1) which had a message on the reverse about how to reduce their oral health risk (Fig. 2). This was explained by the dentist, who ticked the boxes which were most relevant to that patient. In the QLF arm, patients were shown a QLF photograph of their anterior teeth and received a copy printed as a credit card, the back of which again contained messages about how to reduce their risk (Fig. 2). Dentists explained the photograph to patients – explaining any dark (demineralised) or red (mature plaque) areas on the picture.

### Control

Dentists gave usual care (V) with a card containing the checked list of any messages covered (Fig. 2). The reverse of the card was blank.

### Outcomes

#### Primary outcome

The primary outcome was willingness to pay (WTP), an economic measure which quantifies individuals’ preferences for services or goods, increasingly used in the health context [13]. WTP involves identifying the most a consumer would be willing to spend on one unit of a good or service and is viewed as both sensitive enough to detect small changes in preferences as well as practical enough to use in a dental setting [14, 15]. After enrolment, and before their dental check-up, trained dental staff guided participants to use a tablet PC to respond to a WTP task and questionnaire, onto which data were directly entered. All patients were first given descriptions and sample images of the three risk information formats (V, TL and QLF), and asked to rank them in order. Participants were then asked to read a script which emphasised that the next exercise was about value rather than price and which encouraged budget constrained and realistic responses. They were then presented with a series of virtual cards with different values from 50 pence to £150 (randomly ordered) and asked to drag these into one of three boxes: *Would pay/Wouldn’t pay/Not sure* (the shuffled card method). Collection of WTP data also used an incremental approach and follow-up questions to discriminate between protest zeros (unwillingness to engage in the task) and true zeros (value rated as 0) [12, 16]. WTP was elicited first for the least preferred intervention and then the extra WTP for the next preferred was elicited followed by the extra WTP for the most preferred (i.e. WTP for the most preferred was an addition of all three values) [17]. After patients had been randomised and received one of the three types of information during their check-up, they returned to the tablet task, and were reminded of the value they had previously given for the method they received. They were given the option to revise this, with new values collected using an open-ended question.

#### Secondary outcomes (self-perceived oral health and oral health behaviours)

Self-perceived oral health was measured by responses to ‘Would you say your dental health (mouth, teeth and/or dentures) was 1 = very good to 5 = very poor’ [18]. ANCOVA analyses explored whether the information type influenced any change between baseline and follow-up scores. This was done separately for 6-month and 12-month follow-up. Oral health behaviours measured were as follows:

1. Oral hygiene
  - Tooth-brushing frequency: ‘How often do you brush your teeth’ (1 = more than twice a day, 2 = twice a day, 3 = once a day, 4 = less than once a day, 5 = never)
  - Duration of tooth-brushing: ‘How long do you clean your teeth for nowadays?’ (1 = longer than three minutes, 2 = three minutes, 3 = two minutes, 4 = one minute, 5 = shorter than one minute)
2. Dietary sugar
  - Frequency of eating/drinking six items which were cakes or biscuits; puddings or pastries; chocolate or other sweets; fruit juice (not squash); fizzy drinks; soft drinks like squash, measured using a 7-point Likert scale (1 = more than once a day, 2 = once a day, 3 = most days, 4 = at least once a week, 5 = at least once a month, 6 = less than once a month, 7 = never)
  - Frequency of sugar in hot drinks: ‘Do you usually have sugar (not artificial sweetener) in hot drinks like tea and coffee’ (yes/no/I don’t drink hot drinks)
3. Smoking (current smoker (yes/no), excluding e-cigarettes), average number of tobacco items smoked

### Predictor variables

A range of variables were used to explore whether socio-demographic differences explained how people responded to the different types of information. These included the literacy measure Rapid Estimate of Adult Literacy in Medicine, Revised (REALM-R) [19]. A full description of measures used for predictor variables is available [12].

### Sample size

This was calculated to detect significant differences in WTP between the three arms at 80% power with α = 0.05; and based on numbers of standard deviations (SDs) rather than absolute numbers since, in common with other WTP studies, there were no previous valuations of the ‘goods’ (information), on which to base the calculation. Accepting a detectable difference between one-half and one-third of a SD and allowing for around 20% refusal to answer WTP questions (protest responses) gave a figure of 133 in each arm or a total sample size of 400 [12].

### Collection of follow-up data

To collect secondary outcome data, participants were contacted by telephone or e-mail at 6 and 12 months, depending on what contact information the patient gave for this purpose. Patients were lost to follow-up if five contact attempts were unsuccessful. Telephone calls were conducted by a single, trained member of the research team.

### Statistical analysis

**Fig. 3:**
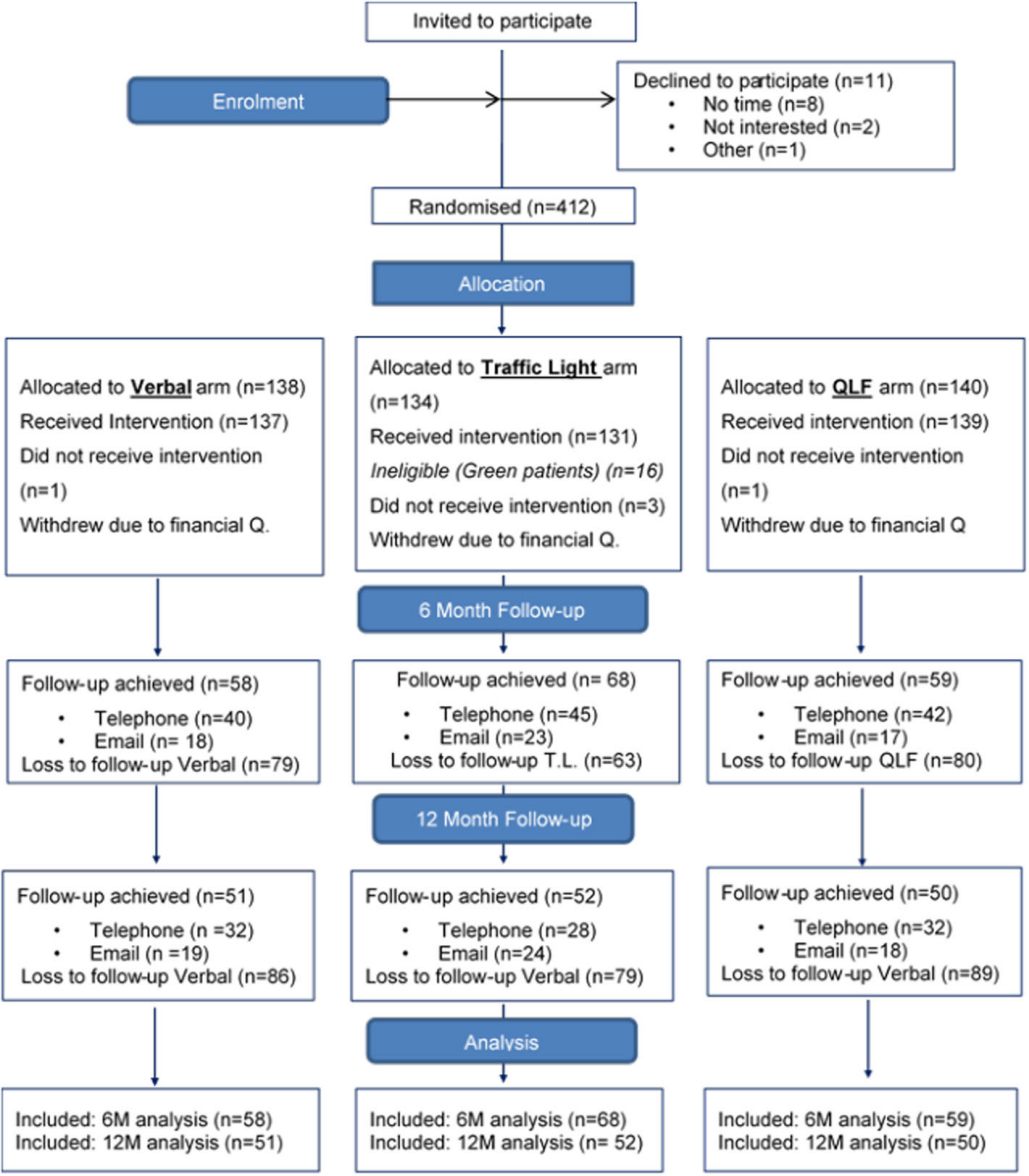
Consolidated Standards of Reporting Trials (CONSORT) diagram with flow of participants through the trial. M months, QLF quantitative light fluorescence

The verbal only and QLF groups did not have the same mechanisms as the TL group for identifying and excluding ‘Green’ cases on the basis of the clinical assessment. This resulted in proportionally more patients who were randomised to the TL arm being withdrawn after allocation, but before the intervention was delivered (Fig. 3). Clinical data involving periodontal status scores were therefore used to identify 12 probable ‘Greens’ in the verbal arm and 15 in the QLF arm. Separate analyses were run and a comparison made between results which included and excluded these 27 participants.

The main analysis first identified proportions favouring each intervention. Zero responses to WTP questions were classified as true or protest zeros based on follow-up questions, and protest zeros were excluded from the analysis. WTP means and medians were compared with a Wilcoxon signed-rank test. Factors affecting both WTP and ranking of preferences were investigated using Tobit regression models. Intervention effects on behavioural outcomes were tested using multivariate generalised linear mixed models, with differences between baseline and 6 months, and between baseline and 12 months, tested in separate analyses. Information type and potential moderating covariate effects (such as gender, age, income, education, IMD, number of teeth, dental attendance and practice attended) were investigated using multivariate analysis of covariance (MANCOVA). Attrition bias at 6 and 12 months was investigated using binomial regression analysis.

## Results

Figure 3 outlines the flow of participants through the trial. Of 423 people recruited, 412 were randomised and completed WTP primary outcome data. Although the sample size calculation identified that 400 participants were necessary to achieve the appropriate level of power, a final eligibility screen identifying patients as ‘Red or Amber’ could only be undertaken by dentists undertaking this clinical assessment during the check-up, which occurred after randomisation. Thus, some patients were excluded from the study at this point. To compensate for a potential loss of power, dental teams were encouraged to recruit beyond their target of 100 patients, leading to 423 instead of 400 patients being recruited in total. The recruitment period was around a year, with the first patient recruited on 17 August 2015 and recruitment ending on 5 September 2016. Data on the revisited WTP response for the intervention they had received were obtained for 391 (94.9%) participants, because of five withdrawals following a baseline personal income question and 16 patients in the TL arm being excluded by dental practices after they judged them to be ‘Green’. Follow-up data were obtained from 185 (44.9%) participants at 6 months and 153 (37.1%) participants at 12 months post recruitment

Table 1 presents participant characteristics and shows that the sample had a higher proportion of females (60%) than males (40%), but a reasonable spread across the socio-economic gradient (about a third had low socio-economic status characteristics). About a quarter were infrequent dental attenders. Although attrition was more likely in lower compared to middle income groups and was associated with greater sugar consumption at baseline, logistic regression analysis showed no significant bias at 6 months overall (Nagelkerke *R*^2^ = 0.075, *p* = 0.146). There was a significant bias at 12 months however, with higher attrition among low-income participants and those with higher baseline sugar consumption (Nagelkerke *R*^2^ = 0.11, *p* = 0.006).

**Table 1:** Participant characteristics at baseline and follow-up

		Baseline		Follow-up	
		Randomised (*n* = 412)	Allocated (*n* = 407)	6-month (*n* = 185)	12-month (*n* = 153)
Gender	Male	166 (40.3%)	164 (40.3%)	79 (42.7%)	66 (43.1%)
	Female	246 (59.7%)	243 (59.7%)	106 (57.3%)	87 (56.9%)
Age category	18–34 years	125 (30.4%)	130 (31.9%)	46 (24.9%)	43 (28.1%)
	35–64 years	214 (51.9%)	205 (50.4%)	102 (55.1%)	77 (50.3%)
	65+ years	70 (17.0%)	70 (17.2%)	36 (19.5%)	32 (20.9%)
	Missing data	3 (0.7%)	2 (0.05%)	1 (0.5%)	1 (0.7%)
Household income	£0–15,599	141 (34.2%)	136 (33.4%)	51 (27.6%)	34 (22.2%)
	£15,600–31,199	142 (34.5%)	142 (34.9%)	72 (38.9%)	65 (42.5%)
	£31,200+	129 (31.3%)	129 (31.7%)	62 (33.5%)	54 (35.3%)
Education	GCSE or less	139 (33.7%)	191 (46.9%)	92 (49.7%)	68 (44.4%)
	A-levels	145 (35.2%)	89 (21.9%)	35 (18.9%)	33 (21.6%)
	Degree+	128 (31.1%)	127 (31.2%)	58 (31.4%)	52 (34.0%)
IMD decile	Low (1–3)	172 (41.7%)	173 (42.5%)	75 (40.5%)	59 (38.6%)
	Medium (4–7)	135 (32.8%)	131 (32.2%)	60 (32.4%)	54 (35.3%)
	High (8–10)	100 (24.3%)	98 (24%)	46 (24.9%)	39 (25.4%)
	Missing	5 (1.2%)	5 (1.2%)	4 (2.2%)	1 (0.7%)
Natural teeth	< 20	71 (17.2%)	68 (16.7%)	28 (15.1%)	25 (16.3%)
	20+	341 (82.8%)	339 (83.3%)	157 (84.9%)	128 (83.7%)
Dental attendance	Infrequent: < 6 in 5 years	96 (23.3%)	97 (23.8%)	42 (22.7%)	32 (20.9%)
	Frequent: 6+ in 5 years	314 (76.2%)	309 (75.9%)	142 (76.8%)	121 (79.1%)
	Missing	2 (0.5%)	1 (0.2%)	1 (0.5%)	

*IMD* Index of Multiple Deprivation

### Preferences

Prior to randomisation, significantly more participants (209, 50.7%) placed verbal information as their greatest preference compared to 58 (14.1%) participants for TL and (145, 35.2%) participants for QLF (*p* < 0.001). All logistic regression models with stepwise elimination on each information type showed that older adults were more likely to prefer verbal only, with differences by practice (in Practice 2, verbal only was especially favoured) (*p* < 0.0001), although a low pseudo-*R*^2^ value (0.102) indicates the model predicted a small proportion of variation in preference. For the TL model, no factors were associated with choosing this as first preference (*p* = 0.49). For the QLF model, being from certain practices increased the likelihood that this was the first preference (*p* = 0.0002; pseudo-*R*^2^ = 0.086), with all other factors not significant. Participants in Practices 2 and 3 were more likely to rank QLF highest, compared with Practice 4.

### WTP

There was a significant difference between verbal and TL (*p* < 0.0001) and between QLF and TL (*p* < 0.0001), but not between verbal and QLF (*p* = 0.41). TL was valued less than either verbal or (QLF, Table 2).

**Table 2:** Mean and median WTP values for each information form

WTP	Median (£)	Interquartile range	Mean	Standard deviation	95% confidence interval
For verbal information (*n* = 227)	20	7–35	30.20	38.87	24.69–34.89
For traffic light information (*n* = 271)	10	2.5–27.5	20.93	29.49	17.46–24.59
For QLF information (*n* = 291)	18.8	5–35	25.52	30.70	21.76–28.81

*QLF* quantitative light fluorescence, *WTP* willingness to pay

Factors affecting WTP were explored using regression models, with WTP for the most preferred intervention (irrespective of intervention) as the dependent variable. In addition to this overall model, an individual model showing WTP for each intervention was developed but was not significantly different in terms of significant variables, so just the overall model is shown here (Table 3). This model shows preferring TL the most, being from Practice 2 and having fewer than 20 teeth were associated with increased WTP. Having a high IMD and a low REALM-R score was associated with decreased WTP.

**Table 3:** Tobit regression model to show predictors of WTP for most preferred intervention

		Coefficient	Standard error	*t*	*p* > *t*	Lower 95% CI	Upper 95% CI
Intervention most preferred	Verbal	Reference					
	QLF	4.87	2.63	1.85	0.066	−0.32	10.06
	Traffic light	9.65	3.81	2.53	0.012	2.15	17.15
Deprivation (IMD decile)	8–10 (high)	−7.14	3.10	−2.31	0.022	−13.24	−1.04
	4–7 (medium)	Reference					
	1–3 (low)	−4.17	2.80	−1.49	0.138	−9.69	1.35
Income per annum	£31,200+	−2.40	2.90	−0.83	0.409	−8.11	3.32
	£15,600–31,199	Reference					
	£0–15,599	−5.03	2.90	−1.74	0.083	−10.71	0.655
Gender	Female	0.24	2.46	0.10	0.924	−4.61	5.08
	Male	Reference					
Education	University degree (high)	0.05	3.30	0.01	0.988	−6.46	6.56
	A-levels (medium)	Reference					
	GCSE or less (low)	3.25	3.16	1.03	0.305	−2.98	9.48
Age	18–34 years	−.75	3.04	−0.25	0.806	−6.73	5.24
	34–64 years	Reference					
	65+ years	−3.63	3.35	−1.08	0.280	−10.23	2.98
Number of teeth	< 20 teeth	6.94	3.51	1.98	0.049	0.032	13.85
	20+ teeth	Reference					
Dental attendance	Infrequent (< 6 times in 5 years)	−0.45	3.02	−0.15	0.881	−6.41	5.50
	Normal attender (6+ in 5 years)	Reference					
Literacy	Low REALM-R	−11.03	5.09	−2.17	0.031	−21.05	−1.00
	Normal/high REALM-R	Reference					
Practice	Practice 1	3.20	3.51	0.91	0.363	−3.72	10.11
	Practice 2	10.14	3.41	2.97	0.003	3.42	16.85
	Practice 3	2.76	3.80	0.73	0.468	−4.72	10.24
	Practice 4	Reference					
Constant		8.42	4.84	1.74	0.083	−1.10	17.95

*n* = 254, Likelihood Ratio of χ^2^ = 32.52 (*p* = 0.0129), pseudo-*R*^2^ = 0.017

*CI* confidence interval, *IMD* Index of Multiple Deprivation, *REALM-R* Rapid Estimate of Adult Literacy in Medicine, Revised, *WTP* willingness to pay

When participants were asked to re-evaluate WTP for only the information type they had received, the median (IQR) WTP changed from £20 (10–30) to £18.8 (10–25) for V; from £6.5 (2–20) to £8 (2–20) for TL; and from £20 (5–40) to £17 (5–30) for QLF—no significant before and after changes were found (*p* < 0.05, Wilcoxon signed-rank test).

### Self-perceived oral health

No intervention group differences were found at 6 months (V 3.84 (SD = 0.81), TL 3.81 (SD = 0.79), QLF 3.93 (SD = 0.79); *p* = 0.389) or 12 months (V 4.06 (SD = 0.68), TL 3.96 (SD = 0.68), QLF 3.92 (SD = 0.70); *p* = 0.758.

### Behaviour change

Using multivariate generalised linear mixed models, no significant effect in oral health behaviours (smoking; brushing frequency; brushing duration; sugar in food; sugar in drinks; sugar added to drinks) was found for any of the information arms between baseline and 6-month follow-up (*F* = 0.84, *p* = 0.432) or between baseline and 12 months (*F* = 1.90, *p* = 0.150). Analysis using MANCOVAs to test whether there were information-type intervention interaction effects with potential moderators (gender, age, income, education, IMD number of teeth, dental attendance and practice attended) showed none, at either 6-month or 12-month follow-up.

## Discussion

This trial was undertaken in NHS dental practices, recruiting patients identified as having moderate or high risk of poor oral health. Three of the four dental practices were in areas rated as in the most deprived 20% of the country. This presented a challenging setting in which to conduct the study and resulted in a retention rate at 6 months of 46%, which is a study limitation. This is not unusual since recruitment rates, retention and compliance with study protocols have been found to be difficult in other general dental practice trials, even in those located in higher socio-economic areas [20, 21]. Relatively poor understanding about research processes, the model of remuneration in dental practices, time pressures and competing priorities (service vs research) make any type of prospective research in dental practices difficult [22]. Involving patients with poor oral health is even more challenging: as experienced in a previous non-randomised study undertaken in Yorkshire involving six practices and 550 patients, and where only 36% had follow-up primary outcome data available at 24 months [23]. In one of these practices, 74% of patients were lost to follow-up, with attrition rates highest for patients with poor oral health [23]. In our study, 34% of participants came from households with less than £14,000 per annum income, so retention rates of 46% at 6 months and 38% at 12 months are not unsurprising. We identified some retention bias at 12 months (low-income patients and those with highest sugar consumption were less likely to be retained) although not at 6 months, and this should be borne in mind when interpreting results.

A strength of the study was its design as a pragmatic trial, to measure effectiveness of the different types of information given and the degree of beneficial effect in real clinical practice, since the study was intended to directly inform policy and practice. Participating dental practices were invited to participate after being randomly selected from a list of NHS practices for the area. This meant that whilst the practices were relatively representative of others, they included practices with limited experience of participating in research studies—a factor shown to make completion of dental practice-based research problematic [20]. There was thus an inevitable trade-off between maximising the external validity of our study and internal validity [24]. Nevertheless, since primary outcome data (WTP) were collected at the first appointment, this had high rates of completion; there was no significant bias in the characteristics of participants retained at 6 months; and our findings still inform health policy as recommended in recent government guidance concerning NHS new dental contract prototype testing in England [25].

The mix of dental practices involved did, however, mean that the study revealed how significant local implementation is in this kind of intervention. Patients in Practice 2 were more likely to place a higher value of willingness to pay for information than other practices, even though this practice was a 95% NHS practice, located in an area among the 10% most deprived in the country. This indicates that while a social gradient in preference for health information may exist [26], clinicians’ approach to the unfolding consultation dialogue may be a significant factor which influences how risk information is valued, and this is an important lesson from this study [27]. Patients in Practice 2 were significantly more likely to put usual verbal advice as their preferred option. Our sample size meant that we were not able to investigate whether this was a practitioner or practice-related effect; but it is possible that a practice-related effect (over and above the communication style of individual practitioners) is important, and this would bear further investigation relevant to implementation of such policies in primary care settings. Delivery system-based factors and practice culture influencing factors such as the time dedicated to giving information relative to other demands of providing care and managing the practice, prevention/disease orientation and level of provider’s postgraduate education and knowledge, are all known to be important in delivery of practice-based prevention programmes [27]. Certainly, a national evaluation of differential impacts on patient access to appointments following implementation of an earlier version of the new NHS dental contract model identified that dental practice-based effects were important [28]. These included factors such as the level of practice buy-in for the new system, staff cohesion and communication within the practice, and whether practice decision-making was generally anticipatory or responsive to occurrences.

Studies of healthcare communication show that patients from higher socio-economic (SES) groups are more engaged and ask more questions in healthcare consultations [29], leading us to hypothesise that WTP for any type of information would increase in line with a social gradient. Our finding that living in a relatively deprived area was predictive of lower WTP for any time of information was therefore expected. Having a low literacy score also reduced WTP for patients’ most preferred type of information. Nevertheless, since socio-demographic characteristics predicted a relatively small proportion of variance in WTP, we would be wise not to presume that patients from more deprived backgrounds all view this in the same way.

Likewise, it is difficult to be conclusive in explaining why the analysis showed that patients who rated TL information as their first preference were willing to pay more for the information (since the model has a low pseudo-*R*^2^ value indicating that only a relatively small proportion of the variance in preference is predicted by the factors included). One hypothesis may be that perhaps patients who prefer TL information are particularly engaged and interested in their oral health (and therefore willing to pay more). So this finding may have more to do with underlying motivation than any particular appeal of a simple visual presentation of risk information as hypothesised at the outset of the study [5, 6], although this needs to be explored further.

## Conclusion

One of the most surprising findings of the study was that the information type most likely to be used in forthcoming reforms to NHS dentistry was the least preferred by patients. The median WTP for TL accompanied by usual verbal advice was found to be half that of usual advice. Where TL was the most preferred option, this was indicative of a higher value being placed on information in general, although reasons for this finding are unclear. Were these the most informed patients, aware of the significance of the system in NHS dentistry? Perhaps significant is that while practice type predicted first preference for both verbal and QLF information, practice type did not predict a preference for TL information. One explanation for this might be that the TL system is effective in standardising patient communication between practices, even if it less preferred than a more tailored discussion with clinicians which contextualises risk information to make it meaningful at a truly personal level [30]. So while the new system, which is a key part of NHS dental contract reforms, may not have much impact as a risk communication tool, promoting patients’ behaviour, it could still have utility as a quality improvement tool for dental practice.

## Data Availability

The datasets generated and/or analysed during the current study are not publicly available to protect the identity of anonymised individuals, but are available from the corresponding author on reasonable request.

## Abbreviations

IMD: Index of Multiple Deprivation
IQR: Interquartile range
QLF: Quantitative light fluorescence
REALM-R: Rapid Estimate of Adult Literacy in Medicine, Revised
SD: Standard deviation
TL: Traffic light
V: Verbal advice
WTP: Willingness to pay
